# Magnetic Resonance Imaging Measurement of Placental Perfusion and Oxygen Saturation in Early-Onset Fetal Growth Restriction

**DOI:** 10.1111/1471-0528.16387

**Published:** 2020-08-05

**Authors:** R Aughwane, N Mufti, D Flouri, K Maksym, R Spencer, M Sokolska, G Kendall, D Atkinson, A Bainbridge, J Deprest, T Vercauteren, S Ourselin, AL David, A Melbourne

**Affiliations:** a Elizabeth Garrett Anderson Institute for Women’s Health, University College London, London, UK; b Department of Medical Physics and Biomedical Engineering, University College London, London, UK; c School of Biomedical Engineering and Imaging, Kings College London, London, UK; d University of Leeds, Leeds, UK; e Medical Physics, University College Hospital, London, UK; f Centre for Medical Imaging, University College London, London, UK; g University Hospital KU Leuven, Leuven, Belgium; h NIHR University College London Hospitals Biomedical Research Centre, London, UK

**Keywords:** Fetal growth restriction, oxygenation, placenta, pregnancy, relaxometry

## Abstract

**Objective:**

We hypothesised that a multi-compartment magnetic resonance imaging (MRI) technique that is sensitive to fetal blood oxygenation would identify changes in placental blood volume and fetal blood oxygenation in pregnancies complicated by early-onset fetal growth restriction (FGR).

**Design:**

Case–control study.

**Setting:**

London, UK.

**Population:**

Women with uncomplicated pregnancies (estimated fetal weight [EFW] >10th centile for gestational age [GA] and normal maternal and fetal Doppler ultrasound, *n* = 12) or early- onset FGR (EFW <3rd centile with or without abnormal Doppler ultrasound <32 weeks GA, *n* = 12) were studied.

**Methods:**

All women underwent MRI examination. Using a multicompartment MRI technique, we quantified fetal and maternal blood volume and feto-placental blood oxygenation.

**Main outcome measures:**

Disease severity was stratified according to Doppler pulsatility index and the relationship to the MRI parameters was investigated, including the influence of GA at scan.

**Results:**

The FGR group (mean GA 27^+5^ weeks, range 24^+2^ to 33^+6^ weeks) had a significantly lower EFW compared with the control group (mean GA 29^+1^ weeks; –705 g, 95% CI –353 to –1057 g). MRI-derived feto-placental oxygen saturation was higher in controls compared with FGR (75 ± 9.6% versus 56 ± 16.2%, *P* = 0.02, 95% CI 7.8–30.3%). Feto-placental oxygen saturation estimation correlated strongly with GA at scan in controls (*r* = –0.83).

**Conclusion:**

Using a novel multimodal MRI protocol we demonstrated reduced feto-placental blood oxygen saturation in pregnancies complicated by early-onset FGR. The degree of abnormality correlated with disease severity defined by ultrasound Doppler findings. Gestational age-dependent changes in oxygen saturation were also present in normal pregnancies.

## Introduction

Placental insufficiency, where the placenta cannot sufficiently supply the oxygen and nutritional demands of the growing fetus, is the most common cause of antenatal stillbirth in developed countries,^1,2^ and is associated with lifelong consequences.^3,4^ Placental development and function are difficult to measure. In clinical practice, indirect placental function is inferred via fetal growth and fetal Doppler ultrasound, looking for evidence of circulatory redistribution secondary to chronic hypoxia.

Placental insufficiency results in fetal (or intrauterine) growth restriction (FGR). According to a Delphi consensus,^5^ FGR is diagnosed when a fetus is small for gestational age, has a small abdominal circumference or an abnormal growth trajectory, with or without abnormal placental and fetal Doppler blood flow;^6,7^ early-onset FGR is defined as being identified before 32 weeks of gestation. It is often challenging to differentiate between the small healthy fetus and those with FGR, as tests based on maternal predictors^8–10^ are so far insufficiently sensitive.

Poor placental development associated with inadequate spiral artery remodelling in early pregnancy is believed to be the underlying causative pathology of early-onset FGR.^11^ Chronic hypoxia is a critical feature,^12,13^ and understanding this pathophysiology is key to timely diagnosis and management of FGR.^12,14–16^ Measurement of fetal oxygen saturation or oxygen exchange may be useful in optimising diagnosis and management of affected pregnancies, to allow estimation of timing of placental failure and to determine the effectiveness of potential treatments on chronic hypoxia.

Using magnetic resonance imaging (MRI), the entire placenta can be imaged at any gestational age. The placenta is often smaller in FGR compared with controls.^17^ Several studies have conducted diffusion-weighted imaging of the growth-restricted placenta.^18–22^ In the placenta, T2 and T2* relaxation time decrease with increasing gestation^23,24^ and are significantly reduced in placentas from FGR pregnancies.^25,26^ In T1-weighted oxygen-enhanced MRI^25^ the absolute signal is significantly lower in FGR pregnancies. The exact physiological alterations that are assessed by placental MRI are not yet well established; there are likely to be distinct flow compartments from the fetal capillaries, trophoblast space and maternal blood pool that have separate MRI diffusion and relaxation properties. By applying a multi-compartment model of placental tissue it may be possible to disentangle the signal from each compartment.

The aim of this study was to apply such multi-compartment MRI to a cohort of pregnancies complicated by early-onset FGR.

## Methods

### Data

The study was approved by the UK National Research Ethics Service and all participants gave written informed consent (London – Hampstead Research Ethics Committee, REC reference 15/LO/1488). Women beyond 24 weeks of gestational age (confirmed by dating scan) with uncomplicated pregnancies and consecutive cases where early-onset FGR was diagnosed were invited to participate. Early-onset FGR was defined here according to a Delphi consensus, as an estimated fetal weight (EFW) <10th centile (using the Hadlock equation^27^ to estimate fetal weight, and Hadlock centile charts with no customisation^27^), with uterine or umbilical artery Doppler pulsatility index (PI) >95th centile^34^, or EFW <3rd centile with or without Doppler ultrasound abnormality before 32 weeks of gestational age.^28,29^ Our control group was defined as women whose fetus had an estimated fetal weight >10th centile. Pregnancies complicated with fetal structural anomalies, aneuploidy or maternal virus infections (cytomegalovirus, toxoplasma, rubella, HIV) were excluded. Women attending anatomy ultrasound scans in the main obstetric ultrasound department were invited to form our control cohort. Pregnant women with maternal medical complications other than pre-eclampsia were also excluded. All women (FGR and control) underwent a detailed ultrasound assessment of fetal structure, size, maternal uterine artery and fetal umbilical artery, middle cerebral artery (and ductus venosus in FGR cases) close to the time of MRI scan (KM/RA) (22/24 participants had an MRI within 3 days of the corresponding ultrasound, the last two within 1week). Pre-eclampsia was defined as persistently raised maternal blood pressure of 140/90 mmHg with significant proteinuria (spot urinary protein to creatinine ratio ≥0.3). Women continued under routine clinical care and were delivered as clinically indicated. Pregnancies were followed up at birth to record birthweight and gestational age at delivery. Placental histological analysis was performed for all cases of FGR using the Amsterdam Placental Workshop Group Consensus Statement for processing and reporting.^30^

### Patient involvement

We involved our Wellcome-Trust-supported Public and Patient Advisory Group from the beginning of our study to inform our ethics applications, ascertain the broad acceptability of MRI imaging, and guide patient and volunteer recruitment.

### MRI

The MRI was performed in unsedated women placed in left lateral tilt to prevent aortocaval compression. Imaging was performed in three dimensions on 1.5T Siemens Avanto, at seven diffusion-weighting *b*-values (0, 50, 100, 150, 200, 400, 600 seconds/mm^2^) and ten echo times (*t*) (81, 90, 96, 120, 150, 180, 210, 240, 270, 300 milliseconds). All echo times were acquired at *b*-value 0, to allow T2 fitting, and all *b*-values at *t* = 96 milliseconds. In addition, data were acquired at *b*-values 50 and 200 for *t* = 81, 90, 120, 150, 180, 210 and 240 milliseconds. Voxel resolution was 1.9 × 1.9 × 6 mm with full placental coverage (26 slices). To minimise the effect of motion we first used an in-house non-rigid registration routine to align all volumes.^31^

**Figure 1:**
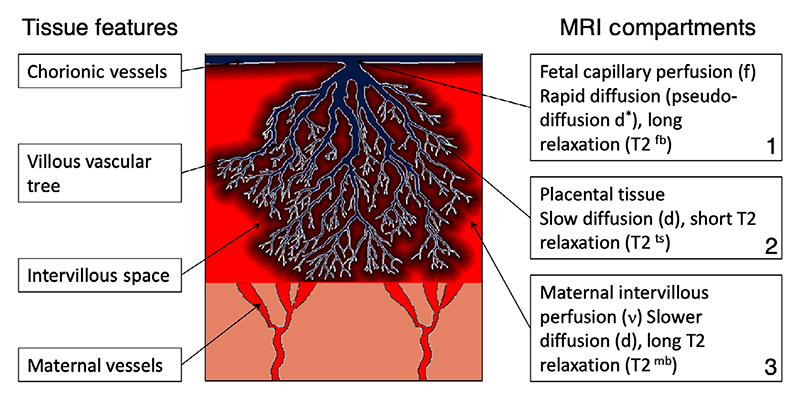
Illustration of the division of the placenta into three different compartments (right hand side) and their respective MRI properties. Key placental features are shown on the left hand side.

DECIDE is a multi-compartment placenta-specific MRI model.^32^ The model combines the T2 relaxometry and diffusion-weighting data above to separate and quantify signals relating to fetal and maternal placental perfusion based upon differences in their respective diffusivity and relaxation (see Supplementary material, Appendix S1). Intracapillary fetal blood has high pseudo-diffusivity, d^*^, and long T2 relaxation time, *T*_2_^fb^ = 1 *=R_2_^fb^* and volume fraction, *f*. Maternal blood with volume fraction *v* is in the intervillous space, as opposed to being intravascular, and therefore has lower apparent diffusivity, *d*, and slow relaxation, R_2_^mb^. Finally, the remaining signal from the tissue has low apparent diffusivity, *d*, and rapid relaxation, *R*_2_^ts^, associated with dense tissue (see Figure 1). The model has previously been described and applied to a small cohort of normal pregnancies.^32^ The DECIDE model was applied for voxel-wise fit of fetal (*f*) and maternal (*v*) perfusion, and feto-placental blood T2 relaxation was converted into blood saturation^33^ (MATLAB R2016b).

This model provides a mechanism to estimate feto-placental blood oxygen saturation.^34^ Given the estimated fetoplacental T2, we can convert this to oxygen saturation values using previously reported data.^32^ Feto-placental oxygen saturation values are estimated by curve fitting to these previously published results (see Supplementary material, Appendix S1).

### Statistics

Data are shown as mean ± standard deviation. Regions of interest were manually defined as pure placental regions within the boundary of the placenta tissue and average parameters were found. Masks were drawn manually over the area of interest (RA) in the registered multiple slices of the two-dimensional stack (itk-SNAP Version 3.2.0, 2014). Statistical analysis was performed with independent Student's *t* test. Where medians and interquartile ranges (IQR) are reported, a Mann-Whitney *U* test was used to determine significance. The FGR cohort was sub-grouped a priori based on the ultrasound findings at the time of the MRI scan into: (1) FGR with uterine and umbilical artery Doppler PI >95th centile (abnormal uterine and umbilical Doppler FGR); (2) FGR with uterine artery Doppler >95th centile and umbilical artery Doppler <95th centile (abnormal uterine Doppler FGR); and (3) FGR with umbilical and uterine Doppler <95th centile (normal uterine and umbilical Doppler FGR).

The MRI parameter comparison between groups was done using Kruskal-Wallis testing to account for the low number of samples, with *post hoc* analysis correcting for the multiple comparisons using Tukey-Kramer correction. We also investigated if any trends existed within the groups by gestational age at scan or birth. Correlations were calculated using Pearson correlation. Significance was set at a threshold of P < 0.05.

*Histograms of regional placental function* In addition to means and standard deviation, we present histogram-driven results as a function of each whole region of interest. This approach avoids the influence of artefacts from amniotic fluid or myometrium and from residual motion. Specifically, we report the inverse of the placental cumulative histogram of feto-placental oxygen saturation, which describes a measure of the fraction of placental tissue above any given oxygen saturation threshold; this is conceptually similar to a continuous ROC curve. We investigate the effect of varying this threshold and find the maximal group separation using a leave-one-out analysis, finding how the maximal separation varies leaving out each subject in turn. This is a post hoc parameter related to the MRI apparent total placental function.

## Results

Twelve women were recruited to each group (see Supplementary material, Table S1) based on effect sizes seen in previous studies.^23,25^ All FGR cases had an estimated fetal weight <3rd centile^35^ (median FGR 681 g [IQR 297 g] versus median control 1358 g [IQR 428 g], *P* = 0.001). There was no difference between groups by mean gestational age at scan (control, 29^+1^ weeks; FGR, 27^+4^ weeks; *P* = 0.15). At the time of MRI of the 12 FGR pregnancies, four had uterine artery Doppler PI >95th centile with umbilical artery Doppler PI >95th centile, four had uterine artery Doppler PI >95th centile with umbilical artery Doppler PI <95th centile and four had normal uterine and umbilical Doppler indices (PIs <95th centile). All women in the control group had normal range umbilical artery Doppler indices, one had uterine artery Doppler PI >95th centile. Middle cerebral artery Doppler values were not significantly different between groups. Ductus venosus was positive in all FGR cases.

Two women in the FGR group had a diagnosis of preeclampsia. Both women had abnormal uterine artery Doppler indices at MRI; one also had an abnormal umbilical artery Doppler at the time of the MRI (26^+1^ weeks), and subsequently had a stillbirth at 27^+1^ weeks of gestation. All women in the control group delivered at term.

Placental histological analysis showed evidence of maternal vascular malperfusion^36^ in four out of the twelve FGR cases (both cases with pre-eclampsia, one case with umbilical and uterine artery Doppler PI >95th centile and one with normal Doppler indices); no pregnancies had evidence of chronic histiocytic intervillositis or chorioamnionitis.

### MRI parameter differences between groups

For the MRI-derived parameters (see Supplementary material, Table S2), we found no difference in mean apparent diffusivity, *d*, (0.0017 ± 0.0001 versus 0.0016 ± 0.0002 mm^2^/ second control versus FGR, *P* = 0.09), maternal perfusion fraction (0.39 ± 0.12 versus 0.32 ± 0.11, *P* = 0.18) or fetal perfusion fraction (0.20 ± 0.03 versus 0.19 ± 0.02, *P* = 0.1) between groups. There was a significant difference in mean placenta T2 relaxation time (204 ± 50 milliseconds versus 143 ± 67milliseconds, *P* = 0.03) and MRI-derived feto-placental blood oxygen saturation estimation (75 ± 9.6% versus 56 ± 16.2%, *P* = 0.02) between groups.

**Figure 2:**
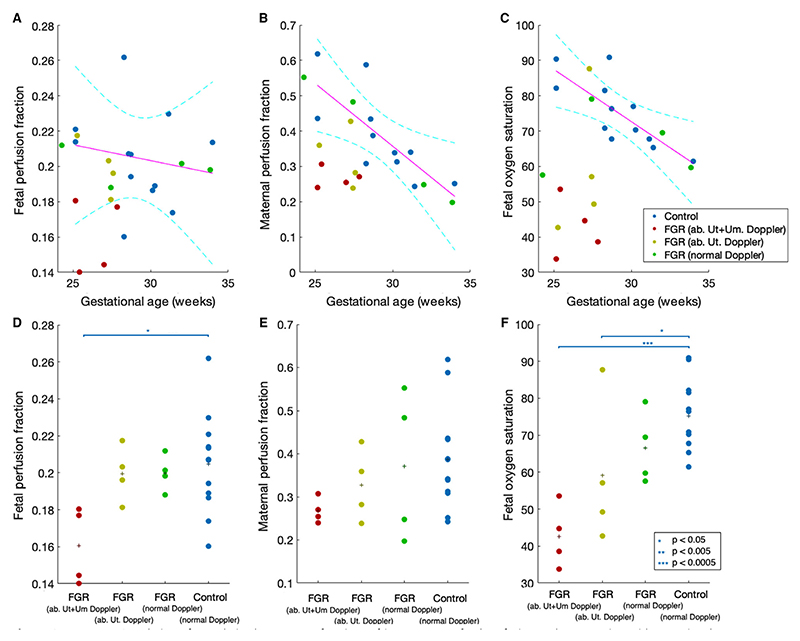
Top row: Correlation of MRI-derived measures of regions of interest mean fetal perfusion and oxygenation with gestational age at scan and group (ab, abnormal; Ut, uterine; Um, umbilical). Trendlines for control group only. Bottom row: Correlation of MRI-derived measures of mean regions of interest fetal perfusion and oxygenation with gestational age at scan and group (ab, abnormal; Ut, uterine; Um, umbilical). Group means shown as plus signs. *P < 0.05, **P < 0.005; ***P < 0.0005.

We studied MRI-derived maternal and fetal perfusion fraction and feto-placental blood oxygen saturation with gestational age (Figure 2 top row). There was no significant correlation between fetal perfusion fraction and gestational age in the control cohort (*r* = –0.16); however, there were significant negative correlations between maternal perfusion fraction (*r* = –0.75) and feto-placental blood oxygen saturation (*r* = –0.80).

Data were sub-grouped based on the ultrasound findings at the time of MRI scan. Pregnancies with abnormal Doppler findings tended to have the greatest difference from the control cohort. Further plots were drawn for each parameter, separating FGR by ultrasound Doppler indicators of disease severity: FGR with uterine and umbilical artery Doppler >95th centile (abnormal uterine and umbilical Doppler FGR, *n* = 4), FGR with uterine artery Doppler >95th centile and umbilical artery Doppler <95th centile (abnormal uterine Doppler FGR, *n* = 4), FGR with umbilical and uterine Doppler <95th centile (normal uterine and umbilical Doppler FGR, *n* = 4), and control (*n* = 12) (Figure 2 bottom row).

There was no significant difference between group means for maternal perfusion fraction (0.27 ± 0.03 versus 0.33 ± 0.08 versus 0.37 ± 0.17 versus 0.39 ± 0.12 for abnormal uterine and umbilical Doppler FGR versus abnormal uterine doppler FGR versus normal doppler FGR versus control, *P* = 0.26); however, for the fetal perfusion fraction there was a significant difference between groups (0.16 ± 0.02 versus 0.20 ± 0.02 versus 0.20 ± 0.01 versus 0.20 ± 0.03, *P* = 0.048 for groups as above) with *post hoc* analysis showing the difference lay between the abnormal uterine and umbilical Doppler FGR group (0.16 ± 0.02) and the control group (0.20 ± 0.03). There was also a significant difference in MRI- derived feto-placental blood oxygen saturation (42+7 ± 8.5 versus 59.2 ± 20.0 versus 66.5 ± 9.9 versus 75 ± 9.6%, *P* = 0.0079, groups as above), with a significant difference between the abnormal uterine and umbilical Doppler FGR group and normal Doppler FGR group (*P* = 0.006) and the control group (*P* = 0.0005) (Figure 2 bottom row).

### Regional placenta function

**Figure 3:**
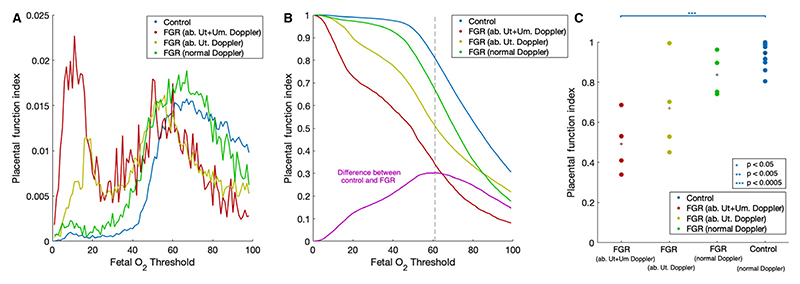
Histograms of placental fetal blood oxygen level. (A) Average histogram for controls (blue, *n* = 12) and FGR groups (abnormal uterine and umbilical fetal Doppler FGR [red], abnormal uterine Doppler FGR [yellow], normal Doppler FGR [green], *n* = 4 per group). (B) Average inverse cumulative histograms for control and FGR groups and difference between all FGR and control (magenta) (ab, abnormal; Ut, uterine; Um, umbilical). (C) Correlation of MRI placental functional index with control and FGR groups with between-group significance marked. Group means shown as plus signs (ab, abnormal; Ut, uterine; Um, umbilical). Placental functional index was calculated as described in the text. *P < 0.05, **P < 0.005; ***P < 0.0005.

Figure 3A shows the average histogram of MRI-derived fetoplacental blood oxygen saturation for each of the control and FGR populations. Figure 3A indicates that the control and FGR distributions have different overall patterns across the placenta. Figure 3B shows the average curve for the control and FGR populations. The difference between these curves – control (*n* = 12) versus all FGR (*n* = 12) – is also shown, with maximal group separation at an oxygen saturation threshold of 61% (Figure 3B). Investigating how this maximal separation varies using a leave-one-out analysis finds the maximal separation to be stable at 60.2% (±2.32%). We use this value to define the Placental Function Index (PFI), which describes the fraction of placental tissue in which the mean feto-placental blood oxygen saturation is >60%. At this level, we found a significant difference in PFI between the control and FGR cohorts (0.94 ± 0.06 versus 0.67 ± 0.22, control versus FGR, *P* = 0.0004) (see Supplementary material, Table S2, Figure 3C).

**Figure 4:**
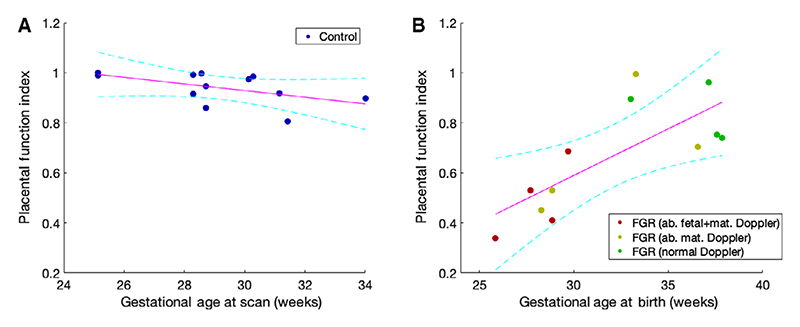
(A) Placenta functional index with gestational age at scan in the control cohort showing a non-significant negative correlation (*r* = –0.53, P = 0.07). (B) Placenta functional index with gestational age at birth in the FGR group cohort, showing a statistically significant correlation (*r* = 0.75, *P* = 0.005) (ab, abnormal; Ut, uterine; Um, umbilical).

The PFI was plotted against gestational age at MRI scan for the control cohort, showing a negative correlation (*r* = –0.53) (Figure 4A). We also found significant differences between groups (0.49 ± 0.15 versus 0.67 ± 0.24 versus 0.84 ± 0.11 versus 0.9 ± 0.06, abnormal uterine and umbilical Doppler FGR versus abnormal uterine doppler FGR versus normal doppler FGR versus control, *P* = 0.006) comparable to the oxygen saturation level in Figure 3(C). Group differences were found between the normal Doppler FGR group and the abnormal uterine and umbilical Doppler group (*P* = 0.005) (Figure 4B). We found a significant positive correlation between the PFI and gestational age at birth in the FGR cohort (*r* = 0.75, *P* = 0.005, Figure 4B).

## Discussion

### Main findings

In this study, we have used a novel multi-modal MRI model to examine the placenta of women with normal fetal size and those affected by early-onset FGR. Our MRI model uses multiple imaging parameters to weight the signal toward blood saturation and perfusion.^32^ We show that our estimates of feto-placental blood oxygen saturation significantly differ between the control and FGR cohorts and that the measure correlated to the disease severity as indicated by the presence of abnormal Doppler indices.

### Strengths and limitations

Our results support our understanding of FGR whereby FGR fetuses with normal Doppler indices may have normal oxygen saturation, whereas FGR fetuses with abnormal Doppler indices are hypoxic. The findings are in keeping with previous studies that directly analysed the oxygen saturation from fetuses with FGR, defined as abdominal circumference <5th centile for gestational age.^13^ The authors found that fetal blood oxygen was significantly lower in FGR versus normally grown fetuses. We defined a PFI between control and FGR placentas to encode the increased spatial heterogeneity seen in FGR placentas. This measure differed according to the severity of the Doppler abnormalities, suggesting that PFI could be a potential marker of disease severity. It may also have a role in the prediction of optimal gestational age at delivery, although this link between gestational age at birth and disease severity is likely to be highly complex. It was also relatively stable in our cohort under a leave-one-out analysis. In the control cohort PFI decreased with increasing gestational age, suggesting that it may also be sensitive to the maturation of placental tissue, where areas within the placenta may no longer be functioning as efficiently. We define this marker here as a *post hoc* parameter, meaning that it is unlikely to eventually be the most robust measurement obtained from this type of data. However, summary measures such as this may in future inform on placental function.

There are other limitations in this work. Our cohort is fairly small, although our results are significant and the effects that we observed are substantive. Our results only apply to early-onset FGR associated with placental insufficiency, and possibly the FGR effects that we observe also have a gestational-age-related component. The MRI parameters will require future validation work with invasive measurement of true oxygen saturation or recourse to sophisticated animal models of growth restriction. It will also be possible to assess reproducibility by longitudinal study of individual pregnancies. The DECIDE model is an early model of placental physiology in MRI and so represents a first approximation of the complexity of placental function. With more advanced image acquisition and analysis it may be possible to develop a more refined model of the MRI signal and so generate a more precise measurement of placental function. Although we did not observe a significant difference in the gestational ages of the control and FGR groups, there were gestational-age dependent changes within the groups that may need to be corrected for, or alleviated by precise and consecutive case–control gestational age matching. Here, the correction of gestational-age-dependent effects in our data would probably enhance the effects that we observe given the linear decrease in measured feto-placental oxygen level with increasing gestational age. Several other contrasts for MRI scans can inform on placental blood flow and function, each with their own advantages and disadvantages.^37^ In our work, we combined diffusion imaging and T2 relaxometry based on results from previous work in pregnancies with small fetuses. Alternative contrasts, for instance T2* relaxometry, could also be incorporated into the type of multicompartment model that we have described. Arterial spin labelled MRI could be used to further establish the contribution of maternal perfusion although this imaging is not without its own challenges.

Our results will eventually help to support the use of MRI for investigating placental insufficiency in less severe phenotypes, which may support a role for MRI in the management of FGR. The cost and complexity of MRI precludes its use as a screening modality; but non-invasive measurement of placental function and feto-placental oxygen level may support prediction of the trajectory of the disease and inform on the optimal timing of delivery, balancing the complications of premature birth against those associated with chronic hypoxia in utero.

### Interpretation

We found no significant difference in apparent diffusivity (d) between control and FGR placenta. This is different from previously published literature, where placental diffusivity values were found to be significantly lower in FGR pregnancies compared to normal controls; however, this significance was found in a much larger cohort^18,19^ and so our results are consistent with the literature.

Previous work has found a difference in total perfusion fraction using a simpler perfusion model (Intra-Voxel Incoherent Motion).^21^ Our model differs from this model by considering T2 relaxation time differences which can bias the measured value of this perfusion fraction.^32,38^ Without this modification, the measured value total perfusion fraction is dependent upon the chosen echo-time^38^ and upon the unknown feto-placental blood saturation.^33^ Specifically, in our model, the value for fetal perfusion is combined with a lower value of T2 to compensate for lower fetal oxygen saturation and is therefore theoretically less biased by these effects, though our results remain consistent with previously published results.^32,38^

We found no difference in MRI-derived maternal or fetal perfusion in the FGR compared with the control cohort. With regard to maternal perfusion the pathophysiology of FGR is thought to be poor maternal placental perfusion secondary to inadequate spiral artery remodelling early in pregnancy.^11^ Our measured values, however, were associated with a large intra-subject variability that may reduce our ability to detect a difference. When categorised according to the severity of the Doppler abnormalities, fetal perfusion in the FGR cases was most reduced when both maternal and fetal Dopplers were abnormal, compared with those FGR cases with less severe or no Doppler abnormalities.

We found a moderate to strong negative correlation between MRI-derived feto-placental blood oxygen saturation and increasing gestational age in the control group. This is in keeping with the findings of previous work^13,39^ from directly sampled blood from the umbilical vessels.

Histograms of the MRI-derived feto-placental blood oxygen level show a bimodal distribution, particularly in those FGR pregnancies compromised by abnormal Doppler indices, with a large proportion of voxels having a fetal blood oxygen saturation <40%, suggesting that the pregnancies with the lowest fraction of placental tissue with mean feto-placental blood oxygen saturation <60% were more compromised. This reflects the heterogeneity seen in structural placental MRI and suggests that these areas of low feto-placental blood oxygen saturation may represent parts of the placenta that are not functioning efficiently with regard to oxygen exchange.

## Conclusion

In summary, using multi-parametric MRI, we have shown a strong link between measurement of feto-placental blood oxygenation and presence of fetal growth restriction. MRI measured feto-placental oxygen saturation correlates with FGR disease severity as indicated by the presence of abnormal umbilical and uterine artery Doppler indices. MRI markers are also correlated to gestational age at delivery in FGR. Multi-compartment MRI may offer the ability to improve our understanding of placental pathophysiology and better define clinical care of growth-restricted babies.

### Disclosure of interests

Dr Atkinson reports grants from Wellcome Trust/UK EPSRC, during the conduct of the study; Dr Spencer reports grants from Wellcome Trust, grants from Radiological Research Trust during the conduct of the study. Prof. David reports grants from Wellcome Trust (203148/Z/16/Z; 203145Z/16/Z; WT101957) and the Engineering and Physical Sciences Research Council (NS/A000049/1; NS/A000050/1; NS/A000027/ 1; EP/L016478/1) during the conduct of the study. The remaining authors have no disclosures. Completed disclosure of interests forms are available to view online as supporting information. Prof. Ourselin reports grants from Wellcome, grants from EPSRC, during the conduct of the study.

### Contribution of authorship

RA contributed to the conception and design of the study, analysis of the data and contributed substantially to the final version of the approved manuscript. NM, DF, KM and RS contributed to the collection of data, analysis of the data and contributed substantially to the final version of the approved manuscript. MS and GK contributed to the collection of data and contributed to the final version of the approved manuscript. DA contributed to the collection of data, analysis of the data and contributed to the final version of the approved manuscript. AB contributed to the collection of data and contributed to the final version of the approved manuscript. JD, TV and SO contributed to the conception and design of the study and contributed to the final version of the approved manuscript. ALD and AM contributed to the conception and design of the study, analysis of the data and contributed substantially to the final version of the approved manuscript.

### Details of ethical approval

The study was approved by the UK National Research Ethics Service and all participants gave written informed consent (London – Hampstead Research Ethics Committee, REC reference 15/LO/1488, original approval 19 October 2015, latest amendment 28 February 2019).

## Supplementary Material

Appendix

Supplementary Tables

## Tweetable abstract

MRI reveals differences in feto-placental oxygen saturation between normal and FGR pregnancy that is associated with disease severity.
