# Supplementary material for: Magnetic Resonance Imaging Measurement of Placental Perfusion and Oxygen Saturation in Early-Onset Fetal Growth Restriction
Source: BJOG. Author manuscript; Available in PMC 2022 Aug 27. (PMC7613436; doi:10.1111/1471-0528.16387)
Supplement: Appendix [file EMS152837-supplement-Appendix.docx]

Appendix S1

**DECIDE MRI estimation of fetoplacental oxygen saturation**

Diffusion-rElaxation Combined Imaging for Detailed Placental Evaluation (DECIDE) is a model for the analysis of structure and function of the placenta using multiple MRI contrast techniques^29^. Multi-modal data of the type described in the methods and an associated computational model allow an interpretation of the diffusion and relaxometry properties in terms of the physiological compartments of the placental tissue.

The DECIDE model assumes that any imaging voxel or region of interest may contain signal from these three compartments in any proportion. Acquiring sufficient images with variable echo time and diffusion-weighting allows us to disentangle the signal contribution of each term and estimate these proportions.

Intracapillary fetal blood has high pseudo-diffusivity, $d^{*}$, and long T2 relaxation time, $T_{2}^{fb}=1/R_{2}^{fb}$ and volume fraction $f$. Maternal blood with volume fraction $\nu$, is in the intervillous space, as opposed to intravascular, and therefore has lower diffusivity $d$, and slow relaxation $R_{2}^{mb}$. Finally, the remaining signal from the tissue has low diffusivity $d$, and rapid relaxation, $R_{2}^{ts}$, associated with dense tissue.

We apply the DECIDE model to fit placental tissue (Eq. A.1) with variables as defined above. All model fitting was done using in-house software developed in MATLAB (The Mathworks Inc., Natick, MA USA).

$\begin{matrix} S(\boldsymbol{b},\boldsymbol{t})=S_{0}[fe^{-\mathbf{b}d^{*}-\boldsymbol{T}_{\boldsymbol{E}}R_{2}^{fb}}+(1-f)e^{-\mathbf{b}d}(\nu e^{-\boldsymbol{T}_{\boldsymbol{E}}R_{2}^{mb}}+(1-\nu)e^{-\boldsymbol{T}_{\boldsymbol{E}}R_{2}^{ts}})] \end{matrix}$ [Eq. A.1]

We apply the same fitting approach described previously so that we may fit fetal blood relaxation $R_{2}^{fb}$, $R_{2}^{mb}$ and $R_{2}^{ts}$ are held fixed at literature values of $(240ms)^{-1}$ and $(46ms)^{-1}$ respectively, whilst all other parameters are fitted. Oxygen saturation can be estimated based on published data^31^ (Eq. A.2).

$T2(s)=a/(1+e^{-g(s-c)})$ [Eq. A.2]

Fitted parameters for this curve, given fractional saturation $s$, are *a=386ms, g=0.36, c=0.88*. We are thus able to find approximate saturation values for each known T2 blood pool.
