# Supplementary material for: Magnetic Resonance Imaging Measurement of Placental Perfusion and Oxygen Saturation in Early-Onset Fetal Growth Restriction
Source: BJOG. Author manuscript; Available in PMC 2022 Aug 27. (PMC7613436; doi:10.1111/1471-0528.16387)
Supplement: Supplementary Tables [file EMS152837-supplement-Supplementary_Tables.pdf]

Table S1. Summary cohort data for control and FGR pregnancies included in the study. IUD: intrauterine death which occurred after MR imaging. Star indicates statistical significance between groups with $p<0.05$.

Subject Characteristics	Control Group (mean (sd, range))	FGR Group (mean (sd, range))
Number (Male/Female)	12 (4/8 M/F)	12 (7/5 M/F)
Estimated Fetal Weight at MRI scan (g)	1451 (476, 798-2384)	746 (344, 342-1484) *
Estimated Fetal Weight centile at MRI scan	55 (23, 19-100)	0.3 (0.7, 0-2) *
Birthweight (g)	3396 (383, 2855 - 4260)	1249 (658, 396 - 2180) *
Gestational age at MRI scan (wks+days)	29+1 (2+4, 25+1 – 34+0)	27+5 (2+5, 24+2 – 33+6)
Gestational age at birth (wks+days) (1 IUD in FGR group)	39+5 (0+6, 38+5 – 41+6)	32+0 (4+3, 27+5 – 37+6) *
Maternal Age at MRI Scan (Yrs)	34.5 (3.0, 29-39)	35.1 (5.6, 24-43)
Mean Uterine Artery Doppler Centile at MRI	32 (31, 2-96)	93 (10, 67-99)
Mean Uterine Artery Doppler PI > 95th centile at MRI	1/12	8/12
Umbilical Artery Doppler Centile at MRI	46 (18, 25-66)	68 (32, 7-99)
Umbilical Artery Doppler PI >95% at MRI	0/12	4/12
Middle Cerebral Artery Doppler PI Centile at MRI	39 (27, 5-85)	30 (30, 1-81)
MCA less than 5th centile	0/12	5/12
Cerebroplacental Ratio (CPR) Centile at MRI	37 (28, 4-85)	24 (34, 1-96)
CPR less than 5th centile	2/12	6/12
Pre-eclampsia	0	2
Iatrogenic preterm delivery	0	7/12
Cord blood arterial pC02	Data Unavailable	7.04 (1.11, 6-8.21)
Cord blood arterial pH	Data Unavailable	7.27 (0.03, 7.22-7.29)
Cord blood venous pH	Data Unavailable	7.32 (0.04, 7.29-7.36)
Admission to NICU (Y/N)	0	9/12

Table S2. Summary of cohort group differences measured using DECIDE MRI. Bold values indicate significant difference ($p<0.05$) between groups.

Imaging Characteristics	Control Group (mean (sd))	FGR Group (mean (sd))	p-value	Confidence Interval	
Placenta Diffusivity, *d* (mm^2s^{-1})	0.0017 (0.0001)	0.0006 (0.0002)	0.09	-0.00002	0.0002
**Placenta Pseudo-Diffusivity, *d** (mm^2s^{-1})**	**0.028 (0.004)**	**0.050 (0.019)**	**0.0009**	**0.034**	**0.01**
**Placenta T2 (ms)**	**204 (50)**	**143 (66.5)**	**0.03**	**8**	**115**
Maternal Perfusion Fraction (*v*)	0.39 (0.12)	0.32 (0.11)	0.18	-0.03	0.16
Fetal Perfusion Fraction (*f*)	0.20 (0.03)	0.19 (0.02)	0.10	-0.003	0.04
**Fetal Blood Oxygen Saturation (%)**	**75 (9.6)**	**56 (16.2)**	**0.02**	**7.8**	**30.3**
**Placenta Function Index (PFI)**	**0.94 (0.06)**	**0.67 (0.21)**	**0.0004**	**0.14**	**0.41**
